# High water intake and low urine osmolality are associated with favorable metabolic profile at a population level: low vasopressin secretion as a possible explanation

**DOI:** 10.1007/s00394-020-02202-7

**Published:** 2020-02-18

**Authors:** Louise Brunkwall, Ulrika Ericson, Peter M. Nilsson, Sofia Enhörning

**Affiliations:** 1 Department of Clinical Sciences, Clinical Research Center, Jan Waldenströms gata 35, Lund University, 214 28 Malmö, Sweden; 2 Department of Endocrinology, Skåne University Hospital, Lund, Sweden

**Keywords:** Copeptin, Glucose, High-density lipoprotein, Urine osmolality, Vasopressin, Water intake

## Abstract

**Purpose:**

Elevated plasma concentration of the vasopressin marker copeptin and low water intake are associated with elevated blood glucose and diabetes risk at a population level. Moreover, in individuals with low urine volume and high urine osmolality (u-Osm), water supplementation reduced fasting plasma (fp) copeptin and fp-glucose. In this observational study, we investigated if low total water intake or high u-Osm correlated with high fp-copeptin and components of the metabolic syndrome at the population level.

**Methods:**

In the population-based Malmö Offspring Study (MOS, *n* = 2599), fp-copeptin and u-Osm from morning urine samples were measured, and diet and total water intake (from beverages and food moisture) was assessed by a 4-day web-based record.

**Results:**

Increasing water intake by tertile was after adjustment for age and sex associated with low fp-triglycerides (*p* = 0.002) and high fp-HDL (*p* = 0.004), whereas there was no association with the other investigated metabolic traits (HbA1c, fp-glucose, BMI or waist circumference). Increasing u-Osm by tertile was, after adjustment for age and sex, associated with high fp-glucose (*p* = 0.007), and borderline significantly associated with high HbA1c (*p* = 0.053), but no association was observed with fp-HDL, fp-triglycerides, BMI or waist circumference. Fp-copeptin concentration correlated significantly with water intake (*r* = − 0.13, *p* < 0.001) and u-Osm (*r* = 0.27, *p* < 0.001). High copeptin was associated with all investigated metabolic traits (*p* < 0.001 for all).

**Conclusion:**

Low concentrations of the vasopressin marker copeptin is linked to high water intake, low u-Osm, and a favorable metabolic profile, suggesting that vasopressin lowering lifestyle interventions, such as increased water intake, may promote metabolic health.

**Electronic supplementary material:**

The online version of this article (10.1007/s00394-020-02202-7) contains supplementary material, which is available to authorized users.

## Introduction

Vasopressin (VP), also called antidiuretic hormone, is released from the posterior pituitary gland mainly in conditions of increased plasma osmolality. In healthy humans, variation of water intake, even within the normal range, is the most well-established factor controlling release of VP. VP regulates water reabsorption through the VP receptor 2 in the kidney. When water intake is low, VP secretion increases, and when water intake is high, VP secretion decreases, hereby keeping the plasma osmolality constant [1].

Elevated plasma copeptin, a reliable marker of VP secretion, has previously been associated with multiple components of the metabolic syndrome [2–4] and with increased risk of diabetes development [5–7]. Previous experimental studies [8, 9] and a Mendelian randomization study [10] points at a causal association between elevated copeptin concentration and increased metabolic risk. It can be speculated that this relationship may be explained by effects mediated by VP receptors expressed in the liver, the pancreas and in the anterior pituitary gland, which potentially could affect glucose metabolism in many different ways by inducing gluconeogenesis, glycogenolysis, glucagon secretion, and increased cortisol release [11–17].

Individuals with low water intake have higher plasma VP and copeptin concentration, and as a result, higher urine osmolality (u-Osm) than individuals with higher water intake [1, 18], whereas increased water intake effectively lowers circulating VP, copeptin, and u-Osm [1, 19]. We recently studied the effect of increased water intake on copeptin concentration and glucose metabolism, and found that individuals with high copeptin concentrations, high u-Osm, and low urine volume (i.e. indices of low water intake) expressed significantly decreased glucagon and copeptin concentrations after 1 week of increased drinking water intake (3 L/day) compared to control week [20]. In another recent study, 6 weeks of increased drinking water intake (1.5 L/day) in low-drinking individuals significantly reduced both fasting copeptin and glucose concentrations [9]. Interestingly, low compared to high water intake, and normal compared to low u-Osm, is associated with elevated blood glucose at the population level [21, 22], which may thus be explained by elevated VP secretion in these conditions [19]. So far, no population-based study has simultaneously investigated reported water intake, u-Osm and copeptin in relation to metabolic syndrome parameters.

In this observational study, we wanted to investigate if low reported total water intake or high u-Osm were associated with components of the metabolic syndrome at the population level, and if low reported total water intake or high u-Osm correlated with high plasma copeptin.

## Subjects and methods

The present study sample consisted of 2599 individuals from the ongoing Malmö Offspring Study (MOS), which is a population-based cohort study in which adult (age > 18 years) children and grandchildren of participants in the Malmö Diet and Cancer–Cardiovascular Cohort were recruited [23, 24]. Participants were invited by letter and visited the research clinic on two occasions. At the first visit, venous blood was drawn after an overnight fast, and anthropometrics were measured. The study participants were instructed on how to collect a morning urine sample and how to register diet and beverage intake at home. The inclusion criterion for MOS is to have a parent or grandparent in the Malmö Diet and Cancer Cohort; no exclusion criteria are used. The ethics committee of Lund University approved the study, which was performed in accordance with the Declaration of Helsinki and its later amendments, and all participants provided written informed consent.

### Anthropometrics

The participants’ height (m) was measured to the nearest centimetre with the participant looking straight ahead and the legs together. Weight (kg) was measured using a calibrated balance or digital scale. Waist circumference (cm) was measured at a level midway between the lowest rib and the *crista iliaca.*

### Urine sampling

The instructions on how to perform urine collections followed a standardized procedure, including a comprehensible video instruction aimed at ensuring accurate collection of urine.

The day before the clinic visit, participants were instructed to empty the bladder before going to bed, and then collect all urine during the night, if any, together with the first morning urine. At the research clinic, the urine sample was stored in a fridge while waiting for transportation to a freezer (− 80 ºC) within 4 h.

### Dietary data

Diet was assessed by “Riksmaten 2010”, a validated 4-day web-based record tool developed by the Swedish National Food Administration and used in the most recent national diet survey in Swedish adults [25]. Participants were instructed to start the diet registration the consecutive day after their first visit to the clinic to get a representation of all weekdays within the cohort. To make the registration as easy and correct as possible, they were provided with a notebook and a photo book with portion sizes. Food intakes were converted into energy, water, and other nutrient intakes using the National food database; *Riksmaten vuxna* 2010 version 10-05-05. Total water intake in g/day included all water in beverages and food moisture. To account for measurement errors and misreporting, intake of fat, protein, and fiber was energy adjusted by dividing the intake in grams with non-alcohol energy intake in kcal [26].

### Laboratory measurements

Fasting plasma copeptin concentration was measured using a KRYPTOR Compact Plus device and commercially available chemiluminescence sandwich immunoassay copeptin ProAVP kit with coated tubes from samples stored at − 80 °C (Thermo Scientific BRAHMS Copeptin proAVP KRYPTOR). Blood glucose was measured using a HemoCue Glucose 201^+^ Analyzer at the research unit. All other plasma and blood laboratory analyses were performed at the University Hospital’s central clinical laboratory in Malmö, and included fasting plasma measurements of lipids, fasting blood HbA_1c_ and fasting plasma creatinine. HbA_1c_ was measured in *n* = 1482 out of 2599 participants as this measurement was added on to the baseline investigation at a later stage.

U-osm in morning urine samples was measured in *n* = 1509 of the study participants at the research unit using an *i*-Osmometer basic (Löser, Germany).

### Statistics

Linear regression models, adjusted for age and sex, were used to analyze the relation between levels of metabolic variables and sex-specific tertiles of water intake, u-osm and copeptin concentrations. Furthermore, water intake analyses were additionally adjusted for physical activity (estimated by a four-grade scale regarding physical activity level during leisure time the past 12 months and ranging from a sedentary lifestyle to regular exercise [27]) and energy-adjusted intake of protein, fat, and fiber. Data from linear regression analyses were expressed as Beta (95% confidence intervals) per unit increase in outcome variable in relation to per tertile increase in water intake, u-osm and copeptin concentrations. Sex-specific tertiles of water intake, u-osm, and copeptin concentrations were used throughout. When Pearson correlations between continuous variables were analysed, copeptin concentrations were transformed using the natural logarithm due to skewed distribution of this variable. When urine osmolality in different tertiles of water intake was analysed, the *p* value was derived from ANOVA.

A two-sided value of *p* < 0.05 was considered statistically significant.

## Results

The present study sample consisted of 2599 individuals who had complete data on anthropometrics and fasting plasma lipids and glucose in the ongoing MOS cohort. They had a mean age of 40 years and 48% were men (Table 1). Out of the 2599 individuals, data on water intake were available in *n* = 1774 participants (Table 2a, Supplemental Table 1), whereas u-osm was measured in *n* = 1509 participants (Table 2b). Plasma copeptin was measured in 2256 participants (Table 2c) and HbA_1c_ was only measured in *n* = 1482. U-Osm and copeptin were markedly lower in women than in men (Table 2b, c), and total water intake was slightly lower in women than in men (Table 2a).

**Table 1:** Study population description—baseline characteristics (*n* = 2599)

Age in years	40 (14)
Sex (proportion of men, *n* (%))	1247 (48)
b-HbA_1c_ in mmol/mol^a^	34.7 (5.9)
p-glucose in mmol/L	5.48 (1.03)
p-triglycerides in mmol/L^b^	0.9 (0.7; 1.3)
p-HDL cholesterol in mmol/L	1.61 (0.48)
BMI in kg/m^2^	25.9 (4.7)
Waist circumference in cm	89.4 (13.8)

Values are presented as mean (s.d.) if not otherwise specified

*BMI* body mass index, *HDL* high-density lipoprotein

^a^Only measured in *n* = 1482

^b^Expressed as median (25th; 75th percentile)

**Table 2:** (a) Total water intake in men and women (*n* = 1774). (b) Urine osmolality in men and women (*n* = 1509). (c) Copeptin concentration in men and women (*n* = 2256)

a			
	Tertiles of water intake	Median	25th; 75th percentile
Men (*n* = 808)	1	1580	1330; 1760
	2	2260	2110; 2430
	3	3110	2840; 3520
Women (*n* = 966)	1	1450	1240; 1640
	2	2090	1940; 2210
	3	2860	2590; 3260

b			
	Tertiles of urine osmolality	Mean	Standard deviation
Men (*n* = 730)	1	410	115
	2	727	81
	3	986	92
Women (*n* = 779)	1	339	76
	2	572	76
	3	896	124

c			
	Tertiles of copeptin concentration	Median	25th; 75th percentile
Men (*n* = 1085)	1	4.20	3.34; 4.97
	2	7.32	6.41; 8.07
	3	12.51	10.48; 15.81
Women (*n* = 1171)	1	2.78	2.30; 3.24
	2	4.59	4.09; 5.18
	3	8.19	6.80; 10.45

Values are given as total water intake (g/days), urine osmolality (mosm/kg H_2_O) and copeptin concentration (pmol/L)

After adjustment for age and sex, there was a significant negative association between increasing total water intake by tertile and triglycerides, and a significant positive association between increasing total water intake by tertile and HDL cholesterol (HDL-C) (Table 3). The associations remained significant after further adjustment for physical activity and dietary intake of fat, protein, and fiber (Table 3). Finally, the results remained similar after additional adjustment for BMI on top of all other covariates (*p* = 0.01 for triglycerides and *p* = 0.007 for HDL-C). There was no association between increasing water intake and fasting plasma glucose, BMI, waist circumference or HbA_1c_ (within the subsample in which Hba_1c_ was available), (Table 3). There was a strong correlation between intake of drinking water and total water (*r* = 0.66, *p* < 0.001).

**Table 3:** Metabolic factors associated with increasing tertiles of total water intake (*n* = 1774)

	Beta (95% CI) in adjusted model^a^	*p*-adjusted model^a^	*p*-adjusted model^b^
HbA_1c_ (mmol/mol)^c^	− 0.11 (− 0.51 to 0.30)	0.61	0.75
Glucose (mmol/L)	− 0.03 (− 0.08 to 0.03)	0.40	0.52
Triglycerides (mmol/L)	− 0.06 (− 0.10 to − 0.02)	0.002	0.04
HDL cholesterol (mmol/L)	0.04 (0.01 to 0.06)	0.004	< 0.05
BMI (kg/m^2^)	− 0.001 (− 0.25 to 0.25)	0.99	0.13
Waist circumference (cm)	0.003(− 0.63 to 0.64)	0.99	0.09

Data expressed as unit change in outcome variable per tertile increase in water intake

*BMI* body mass index, *HDL* high-density lipoprotein

^a^In linear regression adjusted for age and sex

^b^In linear regression adjusted for age, sex, physical activity, energy-adjusted fat intake, energy-adjusted protein intake, and energy-adjusted fiber intake

^c^*n* = 1095

Mean (SD) u-Osm decreased significantly over increasing total water intake tertiles from 719 (247) in tertile 1, to 655 (258) in tertile 2 and 573 (240) in tertile 3 (*p* < 0.001). After adjustment for age and sex, increasing u-Osm by tertile was associated with elevated fasting plasma glucose, and borderline significantly associated with elevated HbA_1c_, (Table 4). The association between increasing u-Osm and elevated fasting plasma glucose remained significant after further adjustment for physical activity (*p* = 0.03) which was analyzed within the majority of individuals in which data on both u-Osm and physical activity level was available (*N* = 1392). No association was seen between increasing u-Osm and HDL-C, triglycerides, BMI or waist circumference (Table 4).

**Table 4:** Metabolic factors associated with increasing tertiles of urine osmolality (*n* = 1509)

	Beta (95% CI) in adjusted model	*p*-adjusted model^a^
HbA1c (mmol/mol)^b^	0.57 (− 0.008–1.16)	0.053
Glucose (mmol/L)	0.08 (0.02–0.14)	0.007
Triglycerides (mmol/L)	0.03 (− 0.02–0.08)	0.24
HDL cholesterol (mmol/L)	− 0.02 (− 0.05–0.007)	0.14
BMI (kg/m^2^)	0.06 (− 0.23–0.34)	0.69
Waist circumference (cm)	0.22 (− 0.51–0.95)	0.56

Data expressed as unit change in outcome variable per tertile increase in urine osmolality

*BMI* body mass index, *HDL* high-density lipoprotein

^a^In linear regression adjusted for age and sex

^b^*n* = 465

There were significant correlations between both copeptin concentration and total water intake (*r* = − 0.13, *p* < 0.001 in all, *r* = − 0.14, *p* < 0.001 in men; *r* = − 0.24, *p* < 0.001 in women) and between copeptin concentration and u-Osm (*r* = 0.27, *p* < 0.001 in all, *r* = 0.20, *p* < 0.001 in men; *r* = 0.24, *p* < 0.001 in women). Furthermore, we found strong positive associations between high copeptin and all investigated metabolic traits (Table 5), which remained significant following additional adjustment for serum creatinine (*p* < 0.001 for all). Finally, we performed multivariate analyses using both copeptin and either u-Osm or total water intake as predictor variables to metabolic traits. These analyses were performed in a limited number of individuals due to lack of overlap between measurement of copeptin, u-Osm, and assessment of dietary data in the cohort. The analyses were tested negatively for multicollinearity (all variance inflation factor (VIF) values obtained were between 1.0 and 1.1). In summary, the results showed that increasing copeptin yielded stronger associations with metabolic traits than increasing u-Osm or total water intake. This was the case in all analyses performed except for the multivariate analysis including u-Osm and copeptin as predictor variables to fp-glucose, in which increasing u-Osm was significantly associated with elevated fp-glucose, whereas copeptin was not (Supplemental tables 2a, b).

**Table 5:** Metabolic factors associated with increasing tertiles of copeptin concentration (*n* = 2256)

	Beta (95% CI) for model 1adjusted model	*p* model 1 adjusted^a^
HbA1c (mmol/mol)^b^	1.00 (0.60 to 1.40)	< 0.001
Glucose (mmol/L)	0.10 (0.04 to 0.15)	< 0.001
Triglycerides (mmol/L)	0.08 (0.05 to 0.12)	< 0.001
HDL cholesterol (mmol/L)	− 0.05 (− 0.07 to − 0.03)	< 0.001
BMI (kg/m^2^)	0.49 (0.27 to 0.72)	< 0.001
Waist circumference (cm)	1.51 (0.93 to 2.09)	< 0.001

Data expressed as unit change in outcome variable per tertile increase in copeptin concentration

*BMI* body mass index, *HDL* high-density lipoprotein

^a^In linear regression adjusted for age and sex

^b^*n* = 1145

## Discussion

The main findings of this observational, population-based study were that high total water intake was associated with a favorable lipid profile independently of physical activity and dietary intake of fat, protein and fiber, and that high u-Osm was associated with elevated fasting glucose. Strong associations between elevated copeptin and all investigated metabolic traits were found, in concordance with previous findings from several other population-based studies [2, 4, 5]. Furthermore, we observed significant correlations between copeptin concentration and water intake and u-Osm, supporting the hypothesis that VP may be an underlying mediator of the associations between measures of water intake and metabolic traits observed in this study.

Vasopressin, measured as the stable marker copeptin, is considered as an established independent risk factor for diabetes, dyslipidemia, the metabolic syndrome, chronic kidney disease, cardiovascular disease, and premature death in the population [3, 5, 28–31]. Furthermore, we previously showed that individuals with high copeptin express a phenotype of high u-Osm and low urine volume; i.e., indices of low water intake [20].

The current study is complementary to and expands our previous finding from an experimental study in humans in which we showed that individuals with low water intake (measured as low 24 h urine volume, high 24 h u-Osm and high copeptin) express a reduction of fasting plasma glucose as a response to increased water intake [9]. In another previous study, we showed that increased water intake in individuals with low water intake was associated with a reduction of the diabetogenic hormone glucagon [20], implicating glucagon as a possible contributing factor to elevated fasting glucose among individuals with low water intake. Furthermore, a beneficial role of increased water intake and decreased circulating VP for metabolic health was recently supported by a study in rats which demonstrated a favorable effect on metabolism when VP was reduced by increased water intake [8]. These data, together with a Mendelian randomization study, in which genetic variation in the human vasopressin gene was recently associated with both elevated copeptin and increased risk of hyperglycemia in men, but not in women [10], provides further support of causality between elevated copeptin, water intake, and metabolic disease.

As far as we know, only one population-based study has previously investigated the impact of water intake on glycemia [21]. These authors stratified the normoglycemic part of a general population into groups of self-reported drinking water intake and found that individuals drinking < 0.5 L of water daily had an increased risk of new-onset hyperglycemia compared to individuals drinking 0.5–1.0 L or > 1.0 L. In the current study, investigating total water intake using a validated dietary assessment method, we were not able to find any link between low water intake and glycemia. However, we found an unfavourable glucometabolic profile in individuals with elevated u-Osm, another measure of water homeostasis of the body, which associates with recorded water intake both in previous studies [22] as well as in our study.

In the present study, women had lower copeptin concentrations than men (Table 2c), replicating previous studies showing sex differences in copeptin concentrations in the population [2, 6]. Furthermore, women had lower u-Osm (Table 2b) which is in line with the previously described lower ability of concentrating urine in women [32]. Around half of the population (38% of the men and 55% of the women) had a daily total water intake above the adequate daily intake, as stated by the European Food Safety Authority, of 2.5 L for men and 2 L for women [33].

### Limitations

Generally, measurement of 24 h u-Osm is considered the most accurate urinary proxy of water intake. In this study, only morning urine samples, which are not as representative of the entire 24-h period, were available. Another limitation is that water intake was self-reported.

It is possible that individuals with elevated plasma glucose have increased u-Osm simply due to elevated glucose levels in urine, which could potentially bias our results. It is normally stated that kidneys conserve glucose until blood glucose reaches over 10 mmol/L [34]. However, the association between increasing u-Osm and fasting plasma glucose was still significant (*p* = 0.02) after excluding all 11 individuals with fasting plasma glucose > 10 mmol/L from the analysis.

In the current study, intake of drinking water was strongly correlated with total water intake and is thus expected to have influenced the observed association between total water intake and a favorable lipid profile. Naturally, other foods and beverages also contribute to total water intake. However, in this study, we were not able to draw any conclusions about which dietary components that contributed most to the association between increased total water intake and beneficial metabolic effects.

The multivariate analyses investigating association between metabolic traits and several hydration markers in the same model (copeptin and either u-Osm or total water intake) were performed in a limited number of individuals due to lack of overlap between measurement of copeptin, u-Osm and assessment of dietary data in the cohort, leading to loss of power and difficulties to interpret the findings. However, our data point out copeptin as the most prominent contributor to the link between hydration markers and metabolic traits, except when it comes to the association with high fp-glucose, where increasing u-Osm seems to be the predominant predictor variable.

Due to the cross-sectional design of this study, it is not possible to draw any conclusions regarding causality.

### Conclusion

Our study extends the previously established association between elevated copeptin and dysregulated lipid and glucose metabolism at a population level by showing that low water intake and high u-Osm are associated with an unfavorable metabolic profile. These findings may be explained by correlations with high concentrations of VP measured with the VP marker copeptin. Our data points at increased hydration as an easily accessible and cost-effective lifestyle intervention for improving metabolic health, and further stresses the need of clinical trials testing the metabolic effects from increased hydration in individuals with low water intake and high copeptin concentrations.

## Electronic supplementary material

Below is the link to the electronic supplementary material.

Supplementary file1 (DOCX 14 kb)

Supplementary file2 (DOCX 15 kb)
