# Supplementary material for: High water intake and low urine osmolality are associated with favorable metabolic profile at a population level: low vasopressin secretion as a possible explanation
Source: Eur J Nutr. 2020 Feb 18;59(8):3715–22. doi: 10.1007/s00394-020-02202-7 (PMC7669756; doi:10.1007/s00394-020-02202-7)
Supplement: Supplementary file 1 — Supplementary file1 (DOCX 14 kb) [file 394_2020_2202_MOESM1_ESM.docx]

**Supplementary table 1 – Covariates across tertiles of water intake (n=1774)**

|  |  | T1 | T2 | T3 | p-value |
| --- | --- | --- | --- | --- | --- |
| Age (years) |  | 39.3 (14.8) | 41.7 (13.6) | 40.2 (13.9) | 0.005 |
| Fat (%) |  | 37.1 (6.7) | 37.4 (6.8) | 37.3 (6.9) | 0.79 |
| Protein (%) |  | 17.8 (4.0) | 17.4 (3.6) | 17.6 (3.9) | 0.14 |
| Fiber (g/MJ) |  | 2.2 (0.7) | 2.3 (0.7) | 2.5 (0.8) | <0.001 |
| Leisure time physical activity (PAL) | Low | 51 (48%) | 30 (28%) | 26 (24%) | <0.001^1^ |
|  | Moderate intensity, not regularily | 229 (37%) | 210 (33%) | 188 (30%) |  |
|  | Moderate intensity regularily | 154 (31%) | 187 (38%) | 151 (31%) |  |
|  | Regular exercise | 158 (29%) | 165 (30%) | 225 (41%) |  |
| Sex-specific tertiles of water intake was used.  Values are presented as mean±s.d. or n (%).  The p-values are derived from ANOVA if nothing else specified.  ^1^ Chi-Square Test | | | | | |
