# Supplementary material for: High water intake and low urine osmolality are associated with favorable metabolic profile at a population level: low vasopressin secretion as a possible explanation
Source: Eur J Nutr. 2020 Feb 18;59(8):3715–22. doi: 10.1007/s00394-020-02202-7 (PMC7669756; doi:10.1007/s00394-020-02202-7)
Supplement: Supplementary file 2 — Supplementary file2 (DOCX 15 kb) [file 394_2020_2202_MOESM2_ESM.docx]

| **Supplemental table 2a. Association between metabolic factors in increasing tertiles of copeptin concentration and total water intake (n=1529)** | | | | |
| --- | --- | --- | --- | --- |
|  | Unit change in outcome variable (Beta (95% CI)) per tertile increase in copeptin concentration | P ^1^  copeptin as predictor variable | Unit change in outcome variable (Beta (95% CI)) per tertile increase in total water intake | P ^1^  total water intake as predictor variable |
| HbA1c (mmol/mol) ^2^ | 0.95 (0.46–1.43) | <0.001 | 0.06 (-0.44–0.55) | 0.82 |
| Glucose (mmol/L) | 0.13 (0.06–0.19) | <0.001 | -0.007 (-0.07–0.06) | 0.84 |
| Triglycerides (mmol/L) | 0.07 (0.03–0.11) | <0.001 | -0.04 (-0.08–0.003) | 0.07 |
| HDL cholesterol (mmol/L) | -0.05 (-0.07–-0.02) | 0.001 | 0.03 (-0.002–0.05) | 0.07 |
| BMI (kg/m^2^) | 0.61 (0.35–0.87) | <0.001 | 0.13 (-0.14–0.39) | 0.34 |
| Waist circumference (cm) | 1.75 (1.07–2.43) | <0.001 | 0.43 (-0.25–1.11) | 0.21 |
| Abbreviations: BMI, body mass index; HDL, high-density lipoprotein.  ^1^ In multivariate linear regression models adjusted for age, sex, tertiles of copeptin and tertiles of total water intake.  ^2^ n=853 | | | | |

| **Supplemental table 2b. Association between metabolic factors in increasing tertiles of copeptin concentration and u-Osm (n=1240)** | | | | |
| --- | --- | --- | --- | --- |
|  | Unit change in outcome variable (Beta (95% CI)) per tertile increase in copeptin concentration | P ^1^  copeptin as predictor variable | Unit change in outcome variable (Beta (95% CI)) per tertile increase in u-Osm concentration | P ^1^  u-Osm as predictor variable |
| HbA1c (mmol/mol) ^2^ | 1.15 (0.19–2.10) | 0.02 | 0.64 (-0.32–1.60) | 0.19 |
| Glucose (mmol/L) | 0.03 (-0.04–0.10) | 0.39 | 0.09 (0.02–0.16) | 0.009 |
| Triglycerides (mmol/L) | 0.09 (0.04–0.14) | <0.001 | -0.03 (-0.08–0.01) | 0.18 |
| HDL cholesterol (mmol/L) | -0.07 (-0.10– -0.04) | <0.001 | 0.005 (-0.03–0.04) | 0.73 |
| BMI (kg/m^2^) | 0.61 (0.29–0.93) | <0.001 | -0.21 (-0.53–0.11) | 0.20 |
| Waist circumference (cm) | 1.67 (0.85–2.50) | <0.001 | -0.54 (-1.38–0.30) | 0.21 |
| Abbreviations: BMI, body mass index; HDL, high-density lipoprotein.  ^1^ In multivariate linear regression models adjusted for age, sex, tertiles of copeptin and tertiles of u-Osm.  ^2^ n=199 | | | | |
